# Reactive oxygen species are signalling molecules for skeletal muscle adaptation

**DOI:** 10.1113/expphysiol.2009.050526

**Published:** 2009-10-30

**Authors:** Scott K Powers, Jose Duarte, Andreas N Kavazis, Erin E Talbert

**Affiliations:** 1 Department of Applied Physiology and Kinesiology, University of FloridaGainesville, FL 32611, USA; 2 CIAFEL, Faculty of Sport, University of PortoPorto, Portugal

## Abstract

Increased reactive oxygen species (ROS) production is crucial to the remodelling that occurs in skeletal muscle in response to both exercise training and prolonged periods of disuse. This review discusses the redox-sensitive signalling pathways that are responsible for this ROS-induced skeletal muscle adaptation. We begin with a discussion of the sites of ROS production in skeletal muscle fibres. This is followed by an overview of the putative redox-sensitive signalling pathways that promote skeletal muscle adaptation. Specifically, this discussion highlights redox-sensitive kinases, phosphatases and the transcription factor nuclear factor-κB. We also discuss the evidence that connects redox signalling to skeletal muscle adaptation in response to increased muscular activity (i.e. exercise training) and during prolonged periods of muscular inactivity (i.e. immobilization). In an effort to stimulate further research, we conclude with a discussion of unanswered questions about redox signalling in skeletal muscle.

The observation that muscular exercise increases the production of free radicals and other reactive oxygen species (ROS) in skeletal muscles was first reported by Davies *et al.* (1982). Paradoxically, it was later discovered that ROS generation also increases in skeletal muscle fibres following prolonged periods of disuse (e.g. immobilization; Kondo *et al.* 1991). Since these early observations, many studies have confirmed that both muscular exercise and long periods of muscle disuse promote ROS production in skeletal muscle fibres (Powers & Jackson, 2008).

During the 1980s and 1990s it was widely assumed that muscular activity- or inactivity-induced ROS production was cytotoxic to skeletal muscle fibres. However, growing evidence indicates that increased ROS production plays an important role in the regulation of signalling pathways that are required to promote skeletal muscle adaptation in response to both exercise and muscle inactivity. The discovery that ROS play an important role in skeletal muscle adaptation to altered activity patterns is an exciting new area of research and is the focus of this review. Specifically, this report provides an overview of our current knowledge about several potential signalling pathways that link ROS to the remodelling that occurs in skeletal muscle during both exercise training and during prolonged periods of muscle disuse. The first segment of this review identifies the potential sites of ROS production in skeletal muscle. This is followed by several important examples of how ROS interact with cellular signalling pathways that regulate gene expression and, therefore, contribute to skeletal muscle remodelling. We then discuss findings which support the notion that cellular ROS production plays an essential role in skeletal muscle adaptation to both exercise and disuse. We conclude with a discussion of the voids in our knowledge about redox control of muscle adaptation in the hope of stimulating future research in this field.

## Sources of ROS production in skeletal muscle

**Figure 1:**
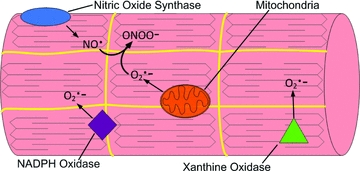
An illustration of the potential sources of reactive oxygen species (ROS) production in skeletal muscleNote that superoxide anions (O_2_^•−^) can be produced at several sites within muscle fibres, including NADPH oxidase, xanthine oxidase and mitochondria. Abbreviations: NO^•^, nitric oxide; O_2_^•−^, superoxide anion; and ONOO^−^, peroxynitrate.

The primary radicals generated in cells are superoxide and nitric oxide, and both can activate a variety of cell signalling pathways (Halliwell & Gutteridge, 2007). Superoxide is generated by the addition of one electron to ground-state oxygen; this can occur via one-electron transfer in the mitochondrion and by several enzymatic systems located within cells. Specifically, superoxide production can occur at multiple locations within the muscle fibre, including the mitochondrion, sarcoplasmic reticulum, transverse tubules, sarcolemma and the cytosol (Fig. 1). The main sites of superoxide production in the mitochondria are complexes I and III of the electron transport chain (Barja, 1999). Furthermore, recent findings indicate that compared with mitochondria from slow type I muscle fibres, mitochondria from fast type II muscle fibres possess unique properties that promote higher levels of ROS production (Anderson & Neufer, 2006). The mechanism(s) to explain these differences remains unknown.

The NADPH oxidases located within the sarcoplasmic reticulum, transverse tubules and the sarcolemma also produce superoxide in skeletal muscle fibres (Powers & Jackson, 2008). For example, it has been reported that the transverse tubule NADPH oxidase produces superoxide which stimulates calcium release from the triads (Hidalgo *et al.* 2006). Nonetheless, limited information currently exists about the regulation of these systems in muscle during exercise or during prolonged disuse.

Evidence also indicates that xanthine oxidase produces superoxide in the cytosol of contracting rat skeletal muscles (Gomez-Cabrera *et al.* 2005). However, compared with rats, human skeletal muscles contain lower levels of xanthine oxidase, and there is continuing debate about whether xanthine oxidase plays an important role in superoxide production in human skeletal muscle (Linder *et al.* 1999; Gómez-Cabrera *et al.* 2003).

The dismutation of superoxide in cells produces hydrogen peroxide (H_2_O_2_), and this process can occur spontaneously or by action of the superoxide dismutases (Halliwell & Gutteridge, 2007). Hydrogen peroxide is a non-radical and a weak oxidant with a relatively long half-life; this long half-life permits diffusion within cells and across cell membranes (Halliwell & Gutteridge, 2007). Furthermore, H_2_O_2_ reacts with many different cellular molecules and can activate a variety of signalling pathways. Collectively, these properties make H_2_O_2_ an important ROS signalling molecule in cells (Veal *et al.* 2007).

Nitric oxide is synthesized from the amino acid l-arginine by three different isoforms of nitric oxide synthase (NOS1, NOS2 and NOS3). Furthermore, a fourth mitochondrial nitric oxide synthase may also exist (Ghafourifar & Cadenas, 2005). Normally, skeletal muscle expresses two of these isoforms (i.e. NOS1 and NOS3; Moylan & Reid, 2007). However, NOS2 can also be expressed in skeletal muscle during inflammatory states (Moylan & Reid, 2007). Nitric oxide is known to have many signalling functions and can readily react with superoxide to form the strong oxidizing agent, peroxynitrite, leading to the depletion of thiol groups in cells (Moylan & Reid, 2007). This modification of cellular thiol groups could alter redox signalling and may play an important role in numerous cell signalling pathways (Jones, 2006). Peroxynitrite formation also reduces the bioavailability of both superoxide and nitric oxide, which could also influence cell signalling events (Halliwell & Gutteridge, 2007; Powers & Jackson, 2008).

## Examples of redox-mediated signalling pathways

Many studies indicate that ROS are important messengers in signalling pathways leading to cellular adaptation (Allen & Tresini, 2000). Recent evidence reveals that ROS signalling contributes to muscle fibre adaptation in response to both increased contractile activity (i.e. muscular exercise) and prolonged periods of muscle disuse (e.g. immobilization). This seemingly contradictory signalling function of ROS is probably due to differences in both the magnitude and the temporal pattern of ROS generation. For example, a moderate increase in skeletal muscle ROS production during a short time period (e.g. minutes) can activate signalling pathways leading to cellular adaptation and protection against future stresses. In contrast, high levels of ROS production over long time periods (e.g. hours) may result in chronic activation of signalling pathways that promote proteolysis and potentially cell death (Ji *et al.* 2006).

How do changes in the redox status of muscle fibres regulate signalling pathways and gene expression? At present, a complete answer to this question is not available. Nonetheless, it is clear that redox signalling can influence numerous transcriptional activators leading to altered gene expression and changes in muscle phenotype. In general, a major mechanism by which redox signalling controls gene expression is via the phosphorylation status of transcriptional activating factors. Indeed, ROS play a key role in controlling the activities of many kinases and phosphatases (Chiarugi & Cirri, 2003; Torres & Forman, 2003). Moreover, ROS are known to activate the transcriptional factor nuclear factor-κB (NF-κB; Kandarian & Jackman, 2006). A brief discussion of these topics follows.

## Regulation of cellular kinases by ROS

Many families of kinases exist in cells, and a detailed discussion of kinases exceeds the scope of this review. Nonetheless, as an illustration of ROS signalling in muscle fibres, we will highlight the mitogen-activated protein kinase (MAPK) family as one of the important links between cellular ROS levels and skeletal muscle adaptation. It is well established that MAPKs play a key role in cell signalling, because the control of numerous cellular signalling pathways is achieved via activation or deactivation of regulatory proteins through phosphorylation (Cuschieri & Maier, 2005). Indeed, the highly conserved MAPK family is one of the major kinase families that regulate the conversion of cell signals into cellular responses. These protein kinases contribute to the regulation of life-and-death decisions made in response to various stress signals (e.g. ROS; Matsuzawa & Ichijo, 2005).

**Figure 2:**
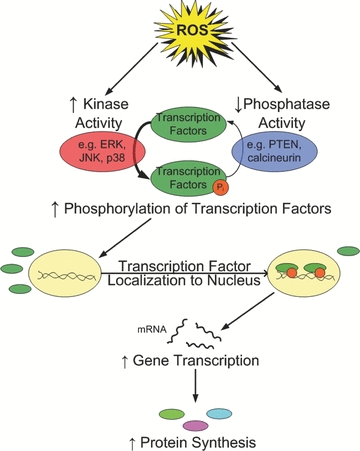
Illustration of the interaction of kinases and phosphatases in the regulation of transcription factors and subsequent protein synthesis in skeletal muscle

All eurkaryotic cells possess multiple MAPK pathways. The three best-characterized MAPK subfamilies are c-Jun N-terminal kinase (JNK), p38 MAPK and extracellular signal-regulated kinase (ERK; Chen *et al.* 2001). All three of these MAPK pathways are structurally similar, but functionally distinct. Importantly, ERK, JNK and p38 MAPK have all been shown to be activated by oxidative stress and could potentially participate in pathways influencing muscle protein breakdown or loss of nuclei via myonuclear apoptosis (Torres & Forman, 2003; Li *et al.* 2005). The classic MAPK activation cascade consists of three sequential intracellular protein kinase activation steps leading to the activation of numerous protein kinases, nuclear proteins and transcription factors, resulting in downstream signal transduction (Chen *et al.* 2001; Cuschieri & Maier, 2005; Fig. 2). A brief overview of the functions of ERK, p38 and JNK follows.

### Extracellular signal-regulated kinase 1/2

The first-identified and best-studied MAPK cascade is the ERK pathway (Cuschieri & Maier, 2005; Meloche & Pouyssegur, 2007). Extracellular signal-regulated kinase is composed of two isoforms, ERK1 and ERK2, collectively referred to as ERK1/2 (Chen *et al.* 2001; Cuschieri & Maier, 2005). Extracellular signal-regulated kinase 1/2 can be activated by a number of mitogens, including epidermal growth factor, platelet-derived growth factor and ROS (Cuschieri & Maier, 2005). The activation of ERK1/2 by oxidative stress is consistent with the hypothesis that low and adequate levels of ROS are mitogenic (Chen *et al.* 2001; Qi & Elion, 2005). Activation of ERK1/2 regulates the transcriptional activity of activator protein-1 (AP-1), c-Myc and the cell survival protein B-cell lymphoma-2 (Bcl-2; Qi & Elion, 2005). Although it is an oversimplification, in general, activation of ERK1/2 promotes cellular adaptations that lead to survival (Chen *et al.* 2001).

### p38 MAPK

p38 is an important MAPK family member that is activated in response to various physiological stresses, such as osmotic stress, endotoxins and ROS (Chen *et al.* 2001). An important link between oxidative stress and p38 activation occurs via apoptosis-stimulating kinase 1 (ASK1; Matsuzawa & Ichijo, 2005). Apoptosis-stimulating kinase 1 is a ubiquitously expressed member of the MAPK family that activates both p38 and JNK by phosphorylating and activating these MAPK kinases. Five isoforms of p38 have been identified: p38α, p38β, p38β2, p38γ and p38δ, and expression of these isoforms varies between tissues. For example, p38α is highly expressed in bone morrow and leukocytes, p38β is expressed in heart and brain, and p38γ is predominantly expressed in skeletal muscle (Cuschieri & Maier, 2005).

Among the phosphorylation targets of p38 are several important transcription factors, including p53, NF-κB and ATF2. Of particular importance to apoptosis is the fact that activation of the tumour suppressor protein p53 results in the expression of the pro-apoptosis protein, Bax. Increased expression of Bax in cells can promote caspase-3 activation via a mitochondrion-mediated pathway (Primeau *et al.* 2002). Moreover, p38 signalling can promote the expression of the muscle-specific E3 ligase atrogin 1 in myotubes (Li *et al.* 2005). Collectively, these findings suggest a potential role for ROS-induced activation of p38 in muscle atrophy. Nonetheless, definitive evidence linking p38 as a causative factor in disuse muscle atrophy does not currently exist.

### c-Jun N-terminal kinase

c-Jun N-terminal kinase (also known as stress-induced kinase) has three isoforms (JNK1, JNK2 and JNK3) that are encoded by three different genes. While JNK1 and JNK2 are ubiquitously expressed, JNK3 is only expressed in brain, heart and testis (Cuschieri & Maier, 2005). c-Jun N-terminal kinase can be activated in response to many of the same stimuli that activate p38, such as osmotic stress, endotoxins and ROS. Moreover, similar to p38, oxidative stress-induced activation of JNK occurs via the ASK1 pathway (Shen & Liu, 2006).

The specific molecular targets of JNK include the transcriptional factors AP-1, p53 and c-Myc and many other non-transcriptional factors, such as Bcl-2 family members. In this regard, there is growing evidence that JNK plays an important role in oxidative stress-mediated apoptosis. Indeed, because ROS themselves are not able to activate caspases, ROS-mediated apoptosis requires another death-signalling pathway, such as JNK (Matsuzawa & Ichijo, 2005; Shen & Liu, 2006).

## Regulation of cellular phosphatases by ROS

Changes in the phosphorylation status of signalling molecules play an important role in the control of cellular adaptation. In this regard, it is important to appreciate that the phosphorylation status of regulatory proteins and/or transcriptional activators is regulated not only by kinase activity but also by changes in phosphatase activity (Fig. 2). In general, phosphatases are divided into two major classes (i.e. serine/threonine phosphatases and phosphotyrosine phosphatases), both of which are known to be redox sensitive in many different cell types, including skeletal muscle. Similar to our discussion of kinases, a detailed discussion of phosphatases exceeds the goals of this review, but examples of important redox-sensitive phosphatases in skeletal muscle are provided in the subsections that follow.

Serine/threonine phosphatases, named for the amino acid residues from which they remove phosphate groups, contain metal ions that are susceptible to oxidation. An important example of a redox-sensitive serine/threonine phosphatase that is crucial to skeletal muscle adaptation is calcineurin. The calcium–calmodulin-dependent calcineurin is involved in both muscle hypertrophy and fibre phenotype transformation, probably via the transcription factor nuclear factor of activated T-cells (NFAT; Dunn *et al.* 1999; Michel *et al.* 2004; Bassel-Duby & Olson, 2006). Calcineurin dephosphorylates NFAT, allowing NFAT to translocate to the nucleus and increase expression of genes that contribute to muscle hypertrophy (Calabria *et al.* 2009). Importantly, oxidation can inhibit calcineurin by blocking its binding domain for calmodulin and thus preventing its activation (Carruthers & Stemmer, 2008). Also, inhibitors of serine/threonine phosphatases have been shown to activate NF-κB, another redox-sensitive transcription factor that will be discussed in the next subsection (Sen *et al.* 1996).

Similar to serine/threonine phosphatases, the phosphotyrosine phosphatases (PTPs) are also susceptible to oxidation-induced inactivation. The PTPs contain a conserved cysteine residue in their active site, and oxidation of this cysteine inactivates the enzyme (Tonks, 2005). A subclass of PTPs, called dual-specificity phosphatases (DUSPs), can remove phosphates from both tyrosine and serine/threonine residues. The DUSPs contain two cysteines in their active sites, leading to inactivation of the enzyme during oxidizing conditions. Of particular importance in skeletal muscle is phosphatidylinositol 3-phosphatase (PTEN). Active PTEN dephosphorylates phosphatidylinositol (3,4,5)-triphosphate, thereby blocking many cell signalling pathways, including Akt activation (Maehama & Dixon, 1998). Exogeneous hydrogen peroxide has been shown to oxidize PTEN, inactivating the enzyme and promoting Akt activation (Leslie *et al.* 2003). Furthermore, the MAPK family is also regulated by a group of DUSPs, which inactivate MAPKs by cleaving their phosphate groups (Camps *et al.* 2000).

## Redox balance and NF-κB activation

Another important link between ROS and skeletal muscle remodelling involves the redox regulation of the NF-κB family of transcriptional activators. Indeed, evidence indicates that the NF-κB pathway is required for skeletal muscle adaptation to both exercise and inactivity-induced atrophy (Kandarian & Stevenson, 2002; Jackman & Kandarian, 2004; Kandarian & Jackman, 2006). In this regard, NF-κB regulates the expression of over 150 genes, and emerging research continues to identify the specific NF-κB targets required for skeletal muscle remodelling (Kramer & Goodyear, 2007).

Nuclear factor-κB comprises a family of five transcription factors (p65, Rel B, c-Rel, p52 and p50). Two of these proteins must dimerize to facilitate binding of NF-κB to DNA and, when activated, NF-κB can promote a wide range of cellular outcomes depending upon the cell type (Jackman & Kandarian, 2004). All five of the NF-κB family members are expressed in skeletal muscle, but recent evidence suggests that the p50–p65 heterodimer is responsible for the majority of NF-κB activity in muscle (Jackman & Kandarian, 2004).

In unstressed cells, the nuclear localization sequence of NF-κB is bound to inhibitory IκB proteins in the cytosol, which prevents dimerization of p50 to p65 and therefore prevents movement into the nucleus. However, increased cytosolic ROS levels can activate IκB-α kinase (IKK), resulting in the phosphorylation of IκB proteins, which initiates ubiquitination and subsequent IκB degradation via the proteasome (Kabe *et al.* 2005). Degradation of IκB removes the inhibition and liberates NF-κB complexes so that dimerization and nuclear translocation can occur (Kabe *et al.* 2005).

Although ROS can promote NF-κB activation and subsequent gene expression, the DNA binding activity of oxidized NF-κB is diminished, suggesting that ROS may also inhibit NF-κB transcriptional activity (Kabe *et al.* 2005). Therefore, while NF-κB was once considered to be a prototypic redox-sensitive transcription factor, the fact that ROS can both promote and inhibit NF-κB transcriptional activation has led to considerable debate regarding the redox control of NF-κB signalling (Pantano *et al.* 2006). Additional work will be required to unravel the uncertainties about the redox regulation of NF-κB in physiologically relevant settings in skeletal muscle.

## Reactive oxygen species and skeletal muscle adaptation to exercise

Physical exercise promotes signalling responses in skeletal muscle leading to alterations in protein synthesis and muscle phenotype. For example, as few as five consecutive days of endurance exercise (i.e. 60 min day^−1^ at 60% of maximal oxygen uptake) promotes an increase in both mitochondrial enzymes and antioxidants in the active skeletal muscles (Vincent *et al.* 2000). The fact that ROS are generated in contracting muscles and many signalling pathways are redox sensitive has raised the question of whether contraction-induced ROS production plays an essential role in skeletal muscle adaptation to aerobic exercise training. The short answer to this question is yes, and evidence to support this position follows.

Davies and colleagues provided the first suggestion that ROS production is a stimulus for skeletal muscle adaptation to exercise training (Davies *et al.* 1982). Since this initial proposal, many studies have supported this concept. For example, abundant *in vitro* studies have demonstrated that exposure of cultured myotubes to oxidants (e.g. hydrogen peroxide) promotes the expression of numerous genes (Li *et al.* 2003; McArdle *et al.* 2004; Hansen *et al.* 2007; Irrcher *et al.* 2009; McClung *et al.* 2009). Specifically, hydrogen peroxide exposure has been shown to augment the expression of key antioxidant enzymes in myotubes (Franco *et al.* 1999). Moreover, a recent study reveals that exposure of myotubes to exogenous hydrogen peroxide increases peroxisome proliferator-activated receptor-γ coactivator-1 protein-α (PGC-1α) promoter activity and mRNA expression (Irrcher *et al.* 2009). Importantly, both effects were blocked when the antioxidant *N*-acetylcysteine was added to the culture medium. It has also been reported that ROS production is a requirement for contraction-induced gene expression of PGC-1α in primary rat muscle cells (Silveira *et al.* 2006). Collectively, these *in vitro* experiments demonstrate that ROS are capable of altering gene expression in cultured muscle cells.

Similar to the aforementioned cell culture studies, numerous reports support the concept that exercise-induced ROS production alters muscle gene expression and contributes to exercise-induced adaptations of skeletal muscle *in vivo*. A common approach in many of these studies is to abolish the signalling effects of exercise-induced ROS production in skeletal muscle by treating animals or humans with antioxidants. For example, two independent reports have demonstrated that exercise-induced expression of heat shock protein 72 in rat skeletal and cardiac muscle is suppressed by antioxidant supplementation (Hamilton *et al.* 2003; Jackson *et al.* 2004). Also, two independent studies have recently concluded that antioxidant supplementation can retard important training adaptations in human skeletal muscle (Gomez-Cabrera *et al.* 2008; Ristow *et al.* 2009). Specifically, Gomez-Cabrera *et al.* (2008) reported that oral administration of vitamin C in humans prevented exercise-induced expression of PGC-1α and mitochondrial biogenesis in skeletal muscle. Furthermore, vitamin C treatment also prevented exercise-induced expression of several antioxidant enzymes in muscle (Gomez-Cabrera *et al.* 2008). Similar results in humans have been reported by Ristow *et al.* (2009). Together, these findings support the conclusion that ROS production plays an essential role in exercise-induced skeletal muscle adaptation.

## Redox signalling and skeletal muscle remodelling during prolonged disuse

As discussed throughout this report, abundant evidence indicates that ROS serve as second messengers in cellular signal transduction pathways that regulate both normal physiological signalling and pathological signalling. This seemingly contradictory signalling function of ROS is probably linked to the level of ROS and the duration of the redox disturbances in the cell. For example, acute increases in ROS promote cell adaptation and survival, whereas chronically elevated levels of ROS production can modulate signalling pathways that lead to proteolysis and cell death (Powers *et al.* 2005, 2007).

It is now well accepted that oxidative stress occurs in skeletal muscle during prolonged disuse and that redox disturbances regulate signalling pathways that contribute to muscle remodelling (e.g. atrophy and loss of myonuclei via myonuclear apoptosis) during these conditions. The first evidence that oxidants play a signalling role in the regulation of disuse muscle atrophy was provided by Kondo *et al.* (1991). This pioneering work revealed that immobilization of skeletal muscles is associated with increased radical production, resulting in oxidative injury in inactive muscle fibres. Importantly, this work also demonstrated that disuse muscle atrophy could be delayed by exogenous antioxidants. These early observations have subsequently been confirmed by others (Appell *et al.* 1997; Betters *et al.* 2004; McClung *et al.* 2007*b*).

The cellular sites of ROS production in skeletal muscle during prolonged inactivity remain a topic of debate. Nonetheless, xanthine oxidase has been shown to be a potential source of oxidant production in rat skeletal muscle during prolonged periods of inactivity (Whidden *et al.* 2009). Moreover, recent evidence indicates that mitochondria may also be an important source of ROS production in skeletal muscle during inactivity (Kavazis *et al.* 2009). In contrast, NOS-mediated production of nitric oxide does not appear to play a role in disuse-induced oxidative stress in skeletal muscle (Van Gammeren *et al.* 2007).

**Figure 3:**
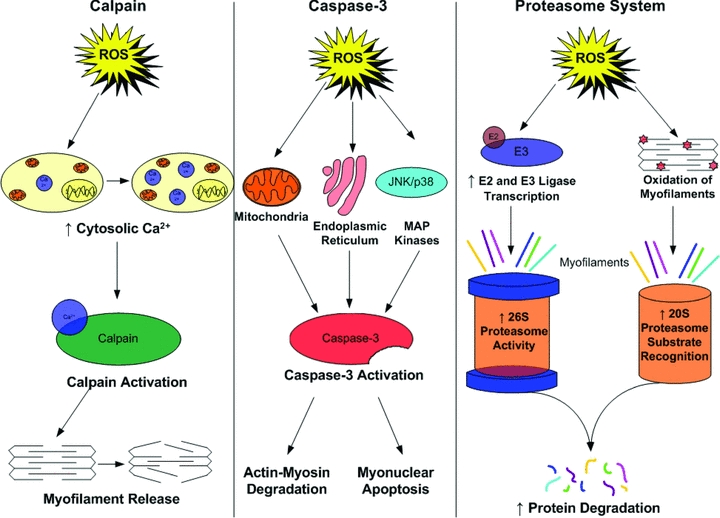
Reactive oxygen species are predicted to activate several proteolytic pathways, including calpains, caspase-3 and the proteasome system in skeletal muscle

How does inactivity-induced oxidative stress in skeletal muscle contribute to muscle atrophy? At present, a complete answer to this question is not available. Nonetheless, it appears that oxidative stress could contribute to disuse muscle atrophy by activation of one or more proteolytic pathways (e.g. calpain, caspase-3 activation and/or the ubiquitin–proteasome pathway; Fig. 3). A brief discussion of these possibilities follows.

### Calpains

Calpains are a family of cellular proteases that are activated by increases in cytosolic levels of free calcium (Goll *et al.* 2003). It is established that calpains are activated during disuse muscle atrophy and that calpain activation may be an initial and required step for disuse muscle atrophy to occur (Goll *et al.* 2003; Maes *et al.* 2007). Although the mechanism responsible for this inactivity-mediated calcium overload is unknown, it is possible that intracellular production of ROS could play a key role in disturbances in calcium homeostasis (Kandarian & Stevenson, 2002). There are at least three mechanisms by which an increase in cellular ROS production can promote increased cytosolic levels of free calcium. First, in cell-free systems, oxidation of the ryanodine-sensitive calcium release channel results in calcium release from the sarcoplasmic reticulum (Powers & Jackson, 2008). Second, high levels of ROS can inhibit sarcoplasmic Ca^2+^-ATPase (i.e. SERCA) activity and retard calcium reuptake into the sarcoplasmic reticulum (Powers & Jackson, 2008). A third potential biochemical mechanism to link oxidative stress with calcium overload is that ROS-mediated formation of reactive aldehydes (i.e. 4-hydroxy-2,3-trans-nonenal) can inhibit plasma membrane Ca^2+^-ATPase activity (Siems *et al.* 2003). Hence, an oxidative stress-induced decrease in membrane Ca^2+^-ATPase activity would impede Ca^2+^ removal from the cell and promote intracellular Ca^2+^ accumulation. Collectively, these mechanisms may act independently or interact to promote oxidative stress-induced calcium overload in cells.

### Caspase-3

Emerging evidence suggests that caspase-3 may play an important role in disuse muscle atrophy (McClung *et al.* 2007*a*). Indeed, caspase-3 activation is capable of promoting degradation of intact actin–myosin complexes, and inhibition of caspase-3 activity has been shown to retard inactivity-induced atrophy and loss of myonuclei due to myonuclear apoptosis (Du *et al.* 2004; McClung *et al.* 2007*a*). Although the control of caspase-3 activity is complex and involves several interconnected pathways, it is feasible that inactivity-associated ROS production in skeletal muscle contributes to caspase-3 activation. Indeed, increased cellular levels of ROS have been reported to activate caspase-3 in a wide variety of cell types, including skeletal muscle (Primeau *et al.* 2002). In this regard, oxidative stress has been postulated to contribute to caspase-3 activation via both mitochondrial and endoplasmic reticulum signalling pathways (Primeau *et al.* 2002). As discussed previously, in the section ‘p38 MAPK’, ROS-induced activation of p38 and/or JNK may play important roles in the redox signalling cascade leading to capase-3 activation.

### Ubiquitin–proteasome system

Finally, several lines of evidence indicate that ROS signalling is involved in the regulation of the ubiquitin–proteasome system. For example, oxidative stress has been shown to stimulate ubiquitin conjugation to muscle proteins through the transcriptional regulation of the enzymes (i.e. E2 and E3 proteins) that conjugate ubiquitin to muscle proteins to promote proteolysis (Li *et al.* 2003). In theory, increased expression of these E2 and E3 proteins in skeletal muscle would lead to accelerated proteolysis via the 26S proteasome. Furthermore, evidence indicates that the 20S (core) proteasome can degrade oxidized proteins without ubiquitination (Grune *et al.* 2003). Therefore, it is likely that oxidant stress can accelerate muscle protein breakdown via both the 20S and the 26S proteasome.

## Conclusions and unanswered questions

Paradoxically, both contractile activity and prolonged periods of muscle disuse promote increased ROS production in skeletal muscle fibres. Importantly, ROS serve as signalling molecules in each of these conditions to influence biochemical pathways and gene expression. Specifically, many signalling molecules are manipulated by ROS, including redox-sensitive kinases, phosphatases and the transcription factor NF-κB. These redox-sensitive molecules act as downstream effectors of ROS and serve as critical signalling events leading to skeletal muscle remodelling in response to increased contractile activity (i.e. physical exercise) or during disuse (e.g. immobilization).

Although progress has been made in understanding the role of ROS as signalling molecules in muscle fibres, many unanswered questions remain. A fundamental query of great importance is ‘how does ROS production in skeletal muscle fibres promote anabolic responses in some conditions (e.g. exercise training), whereas in other states (e.g. disuse), cellular ROS production promotes catabolic signalling?’ Several potential explanations exist for this dichotomy, including differences in the oxidant species produced, divergence in the temporal pattern of ROS production (i.e. acute *versus* chronic ROS production), disparity in the levels of ROS produced and/or differences in the cellular locations of ROS production. Systematic and well-controlled studies are needed to address these important issues.

An on-going limitation in redox biology investigations is the problem of quantifying the levels of different ROS in live cells. Indeed, this restriction has delayed advancements in this field for several years. The creation of sensitive and reliable technique(s) to quantify the levels of ROS in cells or tissues would greatly accelerate progress in oxidative stress research.

Note that much of the evidence linking ROS signalling to muscle adaptation is based on transcriptional changes (i.e. increased or decreased mRNA) in the cell. Future studies should also investigate the role that ROS signalling plays in the rates of translation and in post-translational modifications of proteins.

Hopefully, the questions outlined in this review will stimulate muscle biologists to pursue research in the emerging field of redox signalling. Future technical advances in cell signalling will provide powerful tools for use in redox studies and will supply new opportunities for scientists to address the important questions outlined in this report. Clearly, the field of redox signalling in skeletal muscle is at an exciting stage.
